# SARS-CoV-2-infected adipocytes drive adipose inflammation and hepatocyte lipid accumulation

**DOI:** 10.3389/fimmu.2026.1848780

**Published:** 2026-06-24

**Authors:** Cinthya Alicia Marcela López, Micaela Parra, Rosa Nicole Freiberger, Patricio Jarmoluk, Franco Agustín Sviercz, Jorge Quarleri, M. Victoria Delpino

**Affiliations:** 1 Consejo Nacional de Investigaciones Científicas y Tecnológicas (CONICET), Instituto de Investigaciones Biomédicas en Retrovirus y Sida (INBIRS), Laboratorio de Inmunopatología Viral, Universidad de Buenos Aires (UBA), Buenos Aires, Argentina; 2 Albert Einstein College of Medicine, New York, NY, United States

**Keywords:** adipocytes, hepatocytes, lipid metabolism, lipogenesis, SARS-CoV-2

## Abstract

**Introduction:**

Obesity and fatty liver may worsen COVID-19 outcomes, but the mechanism by which adipose tissue infection contributes to liver injury is unclear. We aimed to determine whether SARS-CoV-2 infection of human adipocytes promotes inflammation and hepatic lipid accumulation, and to identify the underlying mechanisms.

**Methods:**

Mesenchymal stem cell–derived human adipocytes were infected with Wuhan SARS-CoV-2 strain. Cell-surface ACE2 expression was measured in adipocytes. Viral replication and infectious titers were measured, and adipocyte morphology, cytokine/adipokine secretion, and lipid metabolism gene expression were analyzed. Culture supernatants from infected adipocytes were then applied to Huh7.5 hepatocytes to evaluate steatosis and fibrogenic activation.

**Results:**

SARS-CoV-2 productively infected adipocytes, which express cell-surface ACE2, leading to hypertrophy, increased IL-6 secretion, a higher leptin/adiponectin ratio, and lipid droplet accumulation. The infectious virus was released into the supernatants. However, neutralization with anti-Spike antibodies or UV-C inactivation abolished this effect, indicating that hepatocyte lipid accumulation depended on infectious virus rather than on soluble adipocyte-derived mediators alone.

**Discussion:**

Adipose tissue can serve as a source of infectious SARS-CoV-2 that promotes hepatic steatosis and fibrogenic activation, suggesting a mechanism by which obesity and fatty liver may worsen COVID-19 outcomes, although the contribution of residual infectious virus versus adipocyte-derived factors cannot be fully distinguished. At present, the data do not support an independent role for adipocyte-derived soluble mediators in this effect. The study is intended as a mechanistic *in vitro* analysis of adipocyte–hepatocyte crosstalk during SARS-CoV-2 infection.

## Introduction

Coronavirus disease 2019 (COVID-19), caused by SARS-CoV-2, has become a global health crisis since its initial outbreak in Wuhan, China, in December 2019 (1). SARS-CoV-2 is now recognized for its broad organotropism, affecting multiple organs, including the kidneys, liver, heart, gastrointestinal tract, and brain (2). Accumulating evidence indicates that increased adiposity, including obesity and fatty liver disease, is associated with more severe COVID-19 outcomes (3–6). Clinical studies have shown that higher BMI and NAFLD are linked to disease progression and liver injury. In parallel, SARS-CoV-2 can infect human adipose tissue and adipocytes, where it triggers depot-specific inflammatory responses and may contribute to adipose tissue dysfunction (3, 7–9). Autopsy studies have also detected viral RNA in adipose tissue, supporting the concept that fat may act as a viral reservoir and inflammatory amplifier (7, 10, 11). Consistent with this, recent evidence has demonstrated the presence of SARS-CoV-2 RNA in adipocytes from epicardial, visceral, and subcutaneous adipose tissue (7, 12). In addition, infection of mature adipocytes derived from human adipose tissue has revealed the presence of subgenomic RNA, indicating that the virus can replicate in these cells (7). Together, these findings provide a mechanistic basis for the crosstalk between adipose tissue and the liver during COVID-19.

SARS-CoV-2 not only interacts with liver cells but also with adipose tissue. Its ability to directly infect adipose tissue is attributed to the expression of ACE2, the primary receptor for viral entry, on both adipocytes and stromal vascular fraction cells. Lipid accumulation has been observed in SARS-CoV-2-infected cells, indicating that lipid metabolism plays a role in viral pathogenesis (13). Moreover, experimental studies using SGBS cells, as human adipocyte model, have shown that SARS-CoV-2 infection induces transcriptional reprogramming characterized by increased lipid droplet surface area and upregulation of genes involved in inflammation (14).

In the context of obesity, hypertrophied adipocytes display altered endocrine functions, characterized by excessive lipid storage, secretion of proinflammatory adipokines such as TNF-α and IL-6, and impaired insulin sensitivity, all of which contribute to metabolic dysfunction and the development of liver steatosis (15–17). Crosstalk between adipose tissue and the liver is central to this process: proinflammatory signals from adipocytes exacerbate hepatocyte lipid droplet accumulation and promote hepatic inflammation and fibrosis.

In this study, we investigated the impact of SARS-CoV-2 infection on adipose tissue–liver communication. We found that productive infection of adipocytes induces a proinflammatory and lipid-altering phenotype and that infectious material released from these cells can promote lipid accumulation in hepatocytes *in vitro*. These data support a local adipose–hepatocyte crosstalk model.

## Materials and methods

### Cell line culture

The Huh7.5 and Vero E6 cell lines were obtained from ATCC and maintained in Dulbecco’s Modified Eagle Medium (DMEM) supplemented with 10% fetal bovine serum (FBS), 2 mM L-glutamine, 100 U/mL penicillin, and 100 µg/mL streptomycin. Cells were cultured at 37 °C in a humidified incubator with 5% CO_2_.

### Isolation and expansion of mesenchymal stem cells

Umbilical cords were preserved in α-MEM. Fragments (5 mm) were incised to expose Wharton’s jelly, and blood vessels were removed. The fragments were washed with PBS (Sigma-Aldrich) to remove residual blood and placed face down in α-MEM with 10% platelet lysate. Cultures were incubated at 37 °C with 5% CO_2_, with medium changes every 2–3 days. MSC expansion was observed within 10–14 days and continued until passage 2-3. MSC were characterized by CD105, CD73, and CD90 expression, lacking CD45, CD34, CD14, CD19, and HLA-DR (18). For experiments, MSC (up to passage 5) were cultured in α-MEM with 10% heat-inactivated FBS, 100 U/mL penicillin, and 100 µg/mL streptomycin. The Bioethics Committee of the Faculty of Medical Sciences, University of Buenos Aires, Argentina (RESCD-2024-429E) approved this study, with written informed consent obtained from each mother.

### *In vitro* differentiation of MSC into mature adipocytes

MSC were seeded at a density of 5 × 10^4^ cells per well in 24-well plates using α-Minimal Essential Medium (α-MEM; Gibco) supplemented with 2 mM L-glutamine, 10% fetal bovine serum (FBS; Gibco), 100 U/mL penicillin, and 100 μg/mL streptomycin. Adipocyte differentiation was induced using α-MEM with the same base supplements, further enriched with 0.5 mM 3-isobutyl-1-methylxanthine (IBMX), 0.1 µM dexamethasone, 50 μM indomethacin, and 10 µg/mL human insulin (Sigma-Aldrich). Complete differentiation was achieved within 10 days.

All cells used in this study were routinely screened for *Mycoplasma* contamination using the MycoAlert^®^ Mycoplasma Detection Kit (LT07−318, Lonza, Tampa, FL, USA).

### Measurement of ACE2 surface expression in adipocytes

An amount of 1 × 1^6^ adipocytes and Huh7.5 cells (positive control) were washed and incubated with a rabbit primary polyclonal antibody to human ACE2 (PA5.20040, Thermo Fisher Scientific, Waltham, MA, USA) for 1 h on ice. The cells were washed and incubated with a PE-labeled anti-rabbit antibody (ab72465, Abcam, Cambridge, UK). Data were acquired using Full Spectrum Flow Cytometry on the Cytek^®®^ Northern Lights 3000™ (Cytek Biosciences Inc., Fremont, CA, USA) and analyzed with FlowJo v10.6.2 (Ashland, Wilmington, DE, USA).

### SARS-CoV-2 infection of adipocytes

Dr. Sandra Gallego generously provided the Wuhan SARS-CoV-2 strain from the Universidad Nacional de Córdoba, Argentina. The virus was propagated and titrated, in Vero E6 cells, yielding 2.85 × 1^6^ PFU/mL.

Direct exposure of differentiated adipocytes to SARS-CoV-2 was performed at an MOI of 0.01. The infection process involved a 4 h incubation in α-MEM without FBS, followed by 4–5 washes with 1X phosphate-buffered saline (PBS), with the final wash serving as T0, and then culture in supplemented medium.

Culture supernatants from infected and non-infected adipocytes were collected 48 hours post-infection and stored at -70 °C until use. The resulting supernatants were subsequently used to stimulate hepatocytes.

All procedures were conducted in a BSL-3 laboratory at INBIRS, with biological waste autoclaved and incinerated in compliance with institutional safety protocols.

### SARS-CoV-2 infection of Huh7.5 cells

Huh7.5 cells were infected with SARS-CoV-2 at a MOI of 0.01. The infection involved a 4-h incubation in serum-free DMEM, followed by 4–5 washes with 1X PBS. The final wash was designated as T0, and cells were subsequently cultured in supplemented medium.

### SARS-CoV-2 RNA detection and quantification

For SARS-CoV-2, RNA was extracted from culture supernatants using the Chemagic™ Viral DNA/RNA kit special H96 on a Chemagic™ 360 automated platform (PerkinElmer). Quantification was performed with a NanoDrop™ spectrophotometer (Thermo Fisher Scientific), and RNA was normalized for subsequent SARS-CoV-2 detection via RT-qPCR using the DisCoVery SARS-CoV-2 RT-PCR Detection Kit Rox, targeting the ORF1ab and N viral genes according to the manufacturer’s protocol. Viral load in culture supernatants was quantified by interpolating Ct values against a standard curve generated from serial dilutions of a quantified SARS-CoV-2 positive RNA control (GISAID EPI_ISL_420600).

### UV-C irradiation for SARS-CoV-2 inactivation

To evaluate SARS-CoV-2 inactivation, a UV-C light tube emitting at 253.7 nm with an intensity of 500 μW/cm² was positioned 30 cm above culture plates containing the virus (5 mL, 5 × 10^4^ TCID_50_/mL in 10 cm dishes). The plates were exposed to UV-C irradiation for 60 seconds. Complete inactivation was verified for each batch of supernatant before use by assessing the absence of infectivity in Vero E6 cells.

### Evaluation of SARS-CoV-2 infectious particles

To evaluate the release of infectious SARS-CoV-2 particles, Vero E6 cells were used. Vero cells were seeded in 96-well plates and exposed to the culture supernatants under investigation for 1 hour at 37 °C. After this incubation period, the supernatants were removed, and the cells were provided with fresh DMEM containing 2% FBS. The plates were then incubated for an additional 3 days at 37 °C. The infectious titer of each supernatant was determined by the TCID50 assay and expressed as plaque-forming units per milliliter (PFU/mL).

### mRNA extraction and quantitative real-time PCR

Total RNA was isolated from the cells utilizing the Quick-RNA MiniPrep Kit (Zymo Research), adhering strictly to the protocol provided by the manufacturer. From the extracted RNA, 1 µg was used for complementary DNA (cDNA) synthesis through reverse transcription, employing the Improm-II reverse transcriptase enzyme (Promega) to ensure efficient conversion. For gene expression analysis, real-time PCR was performed using SYBR Green, a fluorescent dye that binds to DNA, providing a sensitive and reliable method for detection. The amplification process was carried out on a StepOne Real-Time PCR System (Applied Biosystems), which enabled precise monitoring of the reaction in real time. The following primers pair were used: β-actin sense 5- CCTGGCACCCAGCACAAT-3, antisense 5- CGGGATCCACACGGAGTACT-3; leptin sense 5´-GCTGTGCCCATCCAAAAAGTCC-3′, antisense 5´-CCCAGGAATGAAGTCCAAACCG-3′; adipoQ sense 5´- CAGGCCGTGATGGCAGAGATG-3′, antisense 5´-GGTTTCACCGATGTCTCCCTTAG-3′; fatty acid synthase (FASN) sense 5´-GCGTGGCCGGCTACTCCTAC-3´, antisense 5´-GTGTAGGCCAGTACGTAGGT-3´; adipose triglyceride lipase (ATGL) sense 5´-CAAGCGGAGGATTACTCGCA-3′, antisense 5´-CAAGCGGATGGTGAAGGACA-3′. The amplification cycles for GAPDH were 95 °C for 15 seconds, 55 °C for 30 seconds, and 72 °C for 60 seconds. For leptin and adipoQ were 95 °C for 15 seconds, 59 °C for 30 seconds, and 72 °C for 60 seconds. For FASN and ATGL, were 95 °C for 15 seconds, 60 °C for 30 seconds, and 72 °C for 60 seconds.

All primer sets produced a single product of the correct size. The fold change (relative expression) in gene expression was calculated using the relative quantitation method (2^−ΔΔCt^) (19), and relative expression levels were normalized against β-actin. The standard deviation of Ct values among intra-experiment replicates had to be < 0.5.

### Assessment of lipolytic activity in cultured adipocytes by glycerol release

Glycerol released from differentiated adipocytes was measured using a modified version of the method of Garland and Randle (20). In brief, the culture medium was removed, and cells were rinsed once with phosphate-buffered saline (PBS). Adipocytes were then incubated in PBS containing 2% (w/v) fatty-acid-free bovine serum albumin (BSA) for 6 h at 37 °C in a humidified 5% CO_2_ atmosphere. Following incubation, aliquots of the extracellular medium were collected and centrifuged at 1,000 × g for 5 min at room temperature to remove particulate material. Glycerol in the clarified supernatant was determined with the TG Colour GPO/PAP AA enzymatic assay kit (Wiener Lab) according to the manufacturer’s protocol, and absorbance was read at 505 nm on a microplate reader.

### Quantification of triglyceride content in adipocytes

Differentiated adipocytes were lysed in PBS containing 1% (v/v) Triton X-100, and the resulting lysates were used for triglyceride analysis. Intracellular lipids were extracted directly into the Triton X-100 lysis buffer and enzymatically hydrolyzed to glycerol and free fatty acids using the lipase supplied with the TG Colour GPO/PAP AA kit (Wiener Lab) according to the manufacturer’s instructions. The liberated glycerol was quantified by measuring absorbance at 505 nm on a microplate reader. Triglyceride levels were expressed relative to total protein, which was determined by the Bradford method (Bio−Rad) with bovine serum albumin as the standard.

### SARS-CoV-2 neutralization assay

Neutralization assays against SARS-CoV-2 were performed using anti-Spike protein receptor-binding domain (RBD) F(ab’)2 fragments from hyperimmune equine plasma (Elea Phoenix S.A., Argentina) or anti-snakebite F(ab’)2 fragments as a control (Instituto Biológico Argentino S.A.I.C.). SARS-CoV-2 (Wuhan strain) or UV-inactivated virus was pre-incubated with neutralizing antibody (or control) at 30 µg/mL for 60 minutes at 37 °C. This concentration effectively neutralized the infectivity of a 1 × 1^6^ PFU/mL inoculum, as assessed in Vero E6 cells. Culture supernatants from SARS-CoV-2-infected adipocytes contained 192 ± 24 PFU/mL.

### Assessment of lipid droplet accumulation

Adipocyte differentiation was assessed by lipid droplet accumulation, measured using Bodipy 493/503 (Life Technologies). Cells were cultured on 24-well plates, fixed with 10% formalin for 1 hour, and permeabilized with 0.3% Triton X-100. Lipid droplets were stained with 1 µg/mL Bodipy 493/503 (Invitrogen) and nuclei counterstained with DAPI (Thermo Scientific). Coverslips were mounted in PBS-glycerin (9:1 v/v) and analyzed with a Zeiss LSM 800 confocal microscope. Quantification involved analyzing ten fields per well from three wells per experiment.

### Effects of adipocyte culture supernatants on hepatocyte function

Supernatants from adipocytes infected with the Wuhan SARS-CoV-2 strain were diluted 1:5 in culture medium and used to stimulate hepatocytes for 2 days. Supernatants from non-infected cells were used as controls.

### Statistical analysis

Violin plots were used solely as graphical representations of the data distribution and do not imply non-normality. Normality was assessed using the Shapiro–Wilk test. Statistical analysis was performed using one-way ANOVA, followed by Tukey’s *post hoc* test for multiple group comparisons. Pairwise comparisons were performed using Student’s t-test or the Mann–Whitney test, as appropriate. Each experiment was repeated in triplicate using separate culture preparations on four independent occasions. Data are presented as mean ± SD from 3 to 6 independent biological replicates, each performed in technical triplicate.

Graphical and statistical analyses were done using GraphPad Prism 5.0. Data are presented as mean ± SD, with significance levels indicated as follows: * *p* < 0.01, **p < 0.001, *** *p* < 0.0005, ****p < 0.0001 vs non infected (NI).

## Results

### SARS-CoV-2 infection promotes adipocyte hypertrophy and a proinflammatory profile

**Figure 1:**
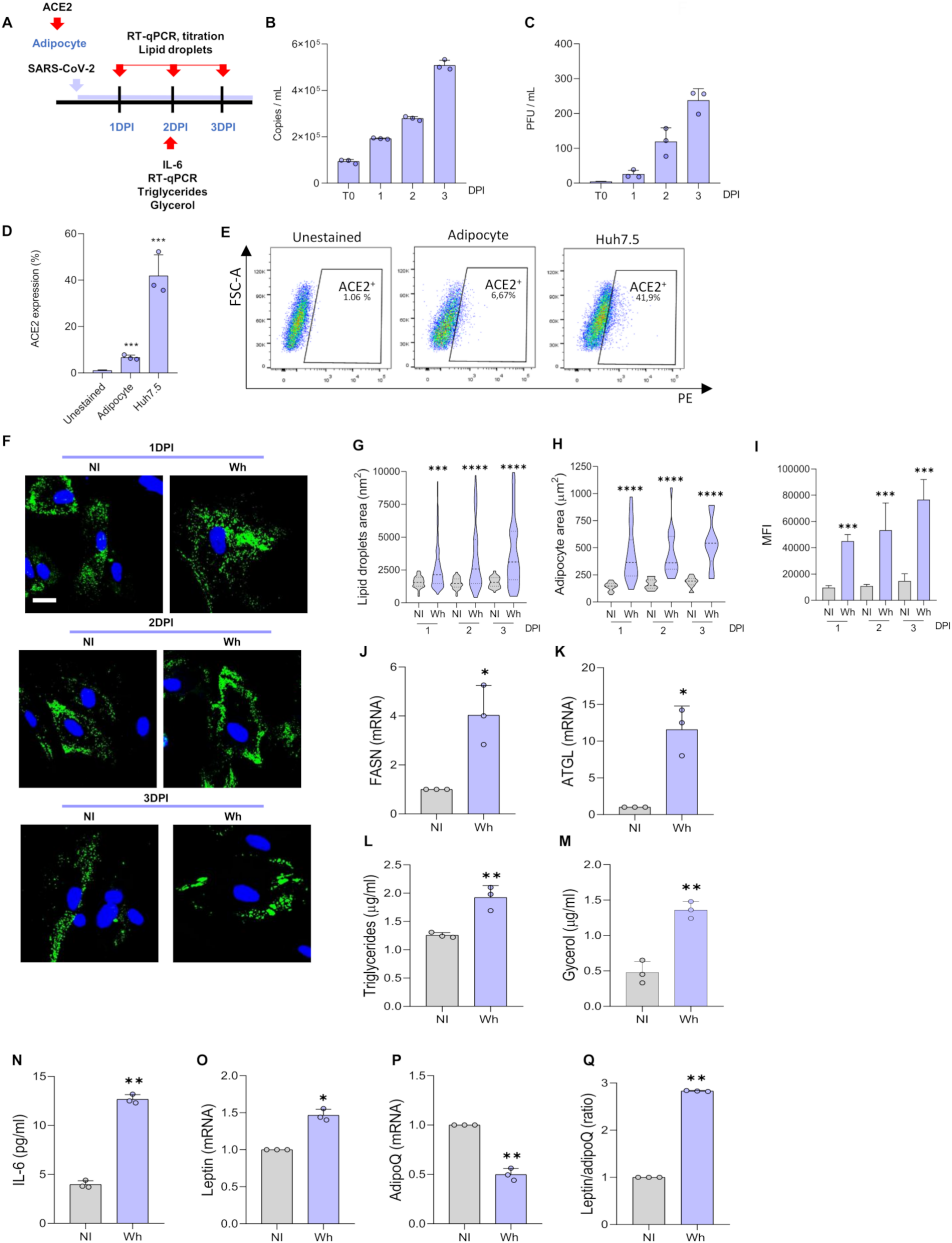
SARS-CoV-2 triggers adipocyte hypertrophy with a proinflammatory phenotype. **(A)** Schematic representation of the experimental timeline. Cell-surface ACE2 expression was measured in adipocytes. Adipocytes were infected with SARS-CoV-2, and viral replication was assessed at 1, 2, and 3 DPI by RT-qPCR and viral titration. At 2 DPI, IL-6 levels were measured in culture supernatants by ELISA, FASN and ATGL expression were quantified by RT-qPCR, and triglyceride and glycerol levels were measured in cell lysates and culture supernatants, respectively. **(B)** Kinetics of SARS-CoV-2 (Wh) replication in adipocytes infected at a multiplicity of infection (MOI) of 0.01. SARS-CoV-2 RNA levels in culture supernatants were quantified by RT-qPCR and expressed as copies/mL. **(C)** SARS-CoV-2 titers in adipocyte culture supernatants, measured as plaque-forming units per milliliter (PFU/mL). **(D)** Evaluation of ACE2 surface expression in adipocytes and Huh7.5 (positive control) measured through flow cytometry **(E)**. Representative histograms obtained by flow cytometry illustrating ACE2 expression as depicted in **(D)**. **(F)** Representative images of lipid droplets stained with Bodipy 493/503. **(G–I)** Quantification of lipid droplets shown in panel D: **(G)** lipid droplet area (W = 0.8691, *p* = 0.82; W = 0.9814, *p* = 0.8491; W = 0.9629, *p* = 0.1171; W = 0.9744, *p* = 0.5191; W = 0.9814, *p* = 0.5217; W = 0.9171, *p* = 0.1667). **(H)** adipocyte area (W = 0.9513, *p* = 0.3605; W = 0.9059, *p* = 0.0622; W = 0.9225, *p* = 0.1626; W = 0.8563, *p* = 0.0862; W = 0.9395, *p* = 0.3429; W = 0.8877, *p* = 0.1888) and **(I)** mean fluorescence intensity (MFI), (W = 0.9643, *p* = 0.6369; W = 0.9887, *p* = 0.7924; W = 0.9868, *p* = 0.7804; W = 0.9231, *p* = 0.4633; W = 0.9973, *p* = 0.8998; W = 0.9643, *p* = 0.6369). **(J)** FASN (W=NA) and **(K)** ATGL (W=NA) transcription measured by RT-qPCR. **(L)** Triglycerides measured in cell lysates (W = 0.9067, *p* = 0.4072; W = 0.9458, *p* = 0.5512). **(M)** Glycerol release was measured in culture supernatants (W = 0.9796, *p* = 0.7262; W = 0.9876, *p* = 0.8872). **(N)** IL-6 secretion was measured in culture supernatant by ELISA (W = 0.8547, *p* = 0.2530; W = 0.9067, *p* = 0.4072). **(O)** Leptin and **(P)** AdipoQ transcription were measured by RT-qPCR (W=NA). **(Q)** Leptin/AdipoQ ratio W=(NA). Non-infected (NI). Days post-infection (DPI), Non applicable (NA). Scale bar: 50 µm. Data are presented as mean ± SD from three independent biological replicates, each performed in technical triplicate. * *p* < 0.01, ** *p* < 0.001, *** *p* < 0.0005, ****p < 0.0001 compared to NI.

Although the precise mechanisms driving fatty liver disease remain to be fully elucidated, emerging evidence implicates dysregulated cross-talk between the liver and adipose tissue (21). In fact, SARS-CoV-2 not only infects respiratory cells but also provokes inflammation in human adipose tissue by targeting both adipocytes and resident macrophages (11, 22–24). To investigate whether SARS-CoV-2 directly alters adipocyte function, we differentiated MSCs into adipocytes and infected them with a multiplicity of infection of 0,01 (Wuhan). Viral replication was monitored over a three-day timeline, illustrated in **Figure 1A**, by quantifying N-gene and ORF1a transcripts in culture supernatants via RT-qPCR, using a post-inoculum wash (T0) as baseline. We observed a steady rise in viral RNA from day 1 through day 3, confirming productive infection (**Figure 1B**), and plaque assays demonstrated the release of virions into the culture supernatants (**Figure 1C**). Therefore, we assessed ACE2 expression, the main receptor for SARS-CoV-2, on the surface of adipocytes. Our results showed that adipocytes express significant levels of ACE2 compared with unstained cells (**Figures 1D, E**).

Concomitant with viral replication, infected adipocytes underwent hypertrophy, characterized by enlarged cells and lipid droplets with a simultaneous increase in mean fluorescence intensity (**Figures 1F–I**). This phenotype was accompanied by upregulation of fatty acid synthase (FASN) and adipose triglyceride lipase (ATGL), increased intracellular triglyceride content, and augmented glycerol release (**Figures 1J–M**). These morphological changes coincided with metabolic and inflammatory alterations: IL-6 secretion was significantly elevated (**Figure 1N**), and the leptin/adiponectin ratio increased (**Figures 1O–Q**). In summary, these data demonstrate that productive SARS-CoV-2 infection induces hypertrophic, proinflammatory reprogramming of adipocytes, creating a dysfunctional state that may promote hepatic steatosis and link viral replication to metabolic and inflammatory disturbances.

### Soluble mediators released by SARS-CoV-2-infected adipocytes contribute to increased lipid droplet accumulation in hepatocytes

**Figure 2:**
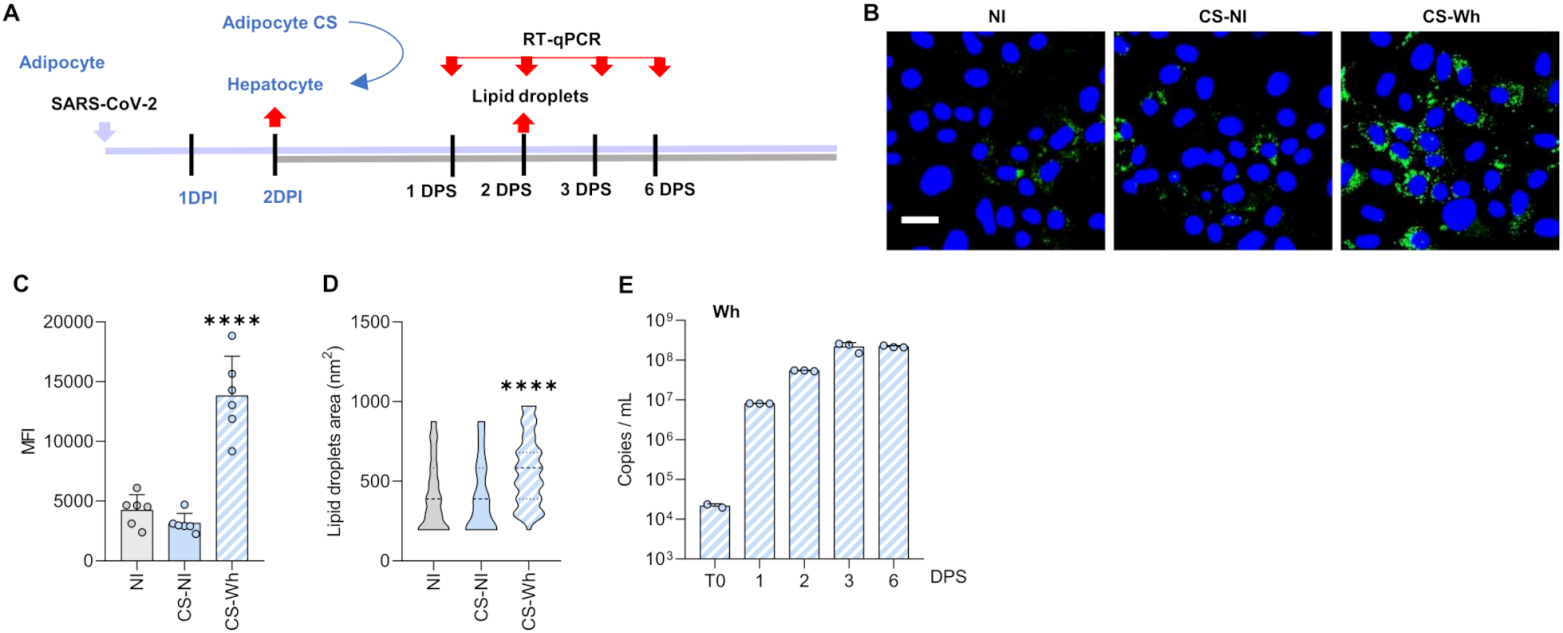
Soluble mediators from SARS-CoV-2–infected adipocytes promote lipid droplet accumulation in hepatocytes. **(A)** Schematic representation of the experimental timeline schedule. Adipocytes were infected, and supernatants were collected at 2 DPI. These culture supernatants were then used to stimulate hepatocytes. Lipid droplets were assessed 2 DPS, and viral replication was measured at 1, 2, 3, and 6 DPS. **(B)** Representative images of lipid droplets from hepatocytes stained with Bodipy 493/503. **(C, D)** Quantification of lipid droplets shown in panel B: **(C)** mean fluorescence intensity (MFI) (W = 0.9345, *p* = 0.6149; W = 0.8024, *p* = 0.0617; W = 0.9961, *p* = 0.9988) and **(D)** lipid droplet area (W = 0.8614, *p* = 0.1501; W = 0.9772, *p* = 0.09; W = 0.9354, *p* = 0.08). **(E)** SARS-CoV-2 (Wh) replication in hepatocytes stimulated with culture supernatants from infected adipocytes. Non-infected (NI). Days post-infection (DPI). Days post-stimulation (DPS). Culture supernatants (CS). Non-significant (NS). Scale bar: 25 µm. Data are presented as mean ± SD from six independent biological replicates for panels C and D, and three independent biological replicates for panel E, each performed in technical triplicate. ****p < 0.0001 compared to CS-NI.

Building on these observations, we next explored the reciprocal impact of infected adipocytes on hepatocyte lipid metabolism. Hepatocytes were treated with culture supernatants from SARS-CoV-2–infected adipocytes, and lipid droplet accumulation was assessed at 2 days post-treatment (**Figure 2A**). We found that supernatants from SARS-CoV-2 (Wuhan)-infected adipocytes not only increased the number of lipid droplets in hepatocytes but also enlarged their size, whereas media from uninfected adipocytes had no effect compared to untreated controls (**Figures 2B–D**). Notably, residual SARS-CoV-2 within the adipocyte secretome retained its infectivity, effectively replicating in hepatocytes (**Figure 2E**). This suggests a pathogenic feed-forward loop that may exacerbate liver–adipose tissue crosstalk during infection.

### Culture supernatants from SARS–CoV–2–infected adipocytes induce hepatic steatosis in a Spike-dependent manner

**Figure 3:**
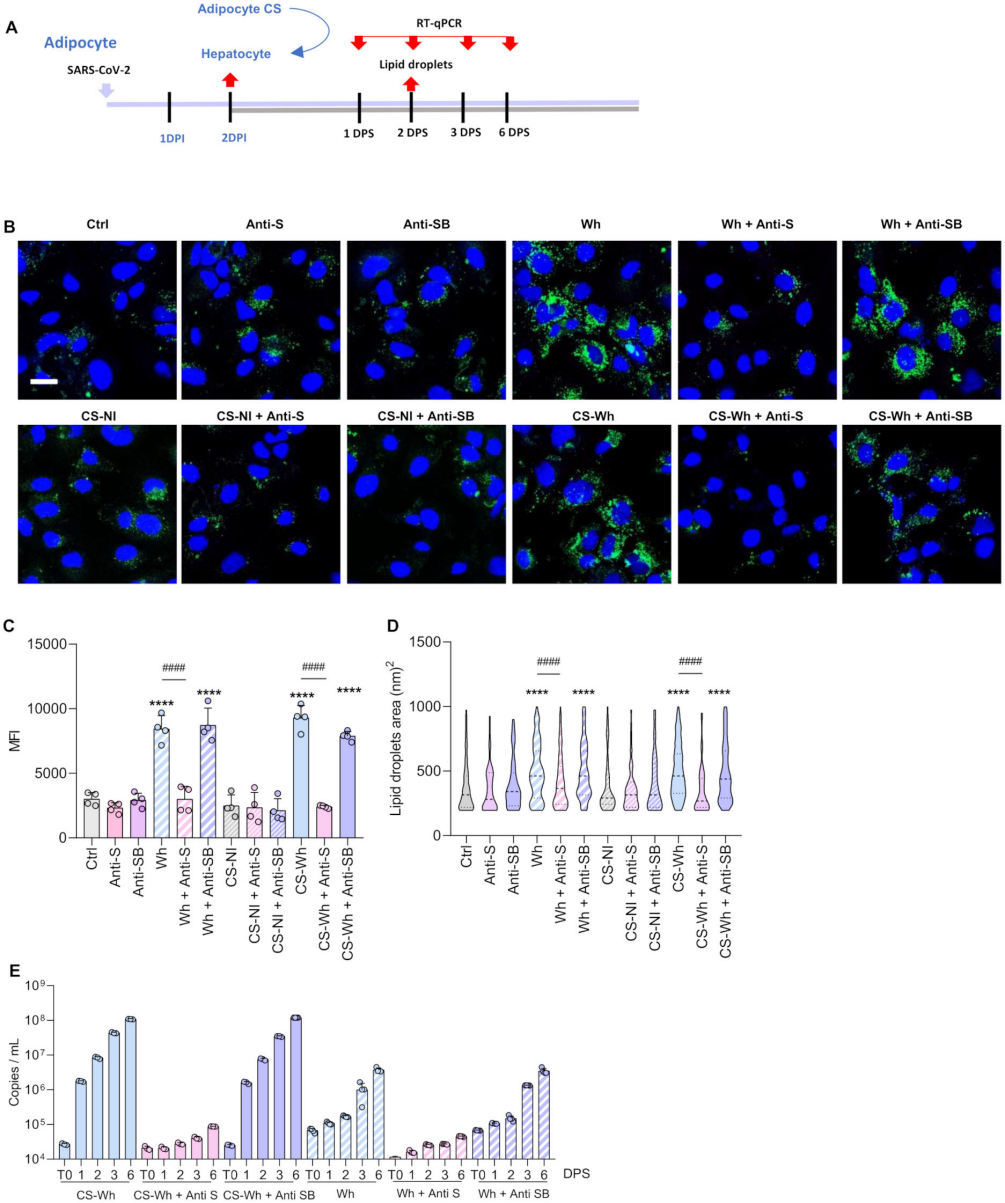
Spike neutralization abrogates lipid droplet accumulation in hepatocytes exposed to supernatants from SARS-CoV-2–infected adipocytes. **(A)** Schematic representation of the experimental timeline. Adipocytes were infected, and supernatants were collected at 2 DPI. These culture supernatants were then used to stimulate hepatocytes in the presence of either an anti-Spike (anti-S) antibody or an irrelevant control antibody against snakebite venom (anti-SB). Lipid droplets were assessed at 2 DPS, and viral replication was measured at 1, 2, 3, and 6 DPS. **(B)** Representative images of lipid droplets in hepatocytes stimulated with culture supernatants from SARS-CoV-2–infected adipocytes pre-incubated for 1 h at 37 °C with either an anti-S antibody or an irrelevant control antibody against anti-SB. Lipid droplets were stained with Bodipy 493/503. **(C, D)** Quantification of lipid droplets shown in panel B: **(C)** mean fluorescence intensity (W = 0.8619, *p* = 0.2671; W = 0.8965, *p* = 0.4141; W = 0.9987, *p* = 0.9960; W = 0.9818, *p* = 0.9127; W = 0.7895, *p* = 0.0846; W = 0.8844, *p* = 0.3579; W = 0.9024, *p* = 0.4431; W = 0.9437, *p* = 0.6770; W = 0.8294, *p* = 0.1663; W = 0.9381, *p* = 0.6431; W = 0.9466, *p* = 0.6950; W = 0.9670, *p* = 0.8226) and **(D)** lipid droplet area (W = 0.9425, *p* = 0.0823; W = 0.9409, *p* = 0.0739; W = 0.8565, *p* = 0.0921; W = 0.9445, *p* = 0.7329; W = 0.8828, *p* = 0.3610; W = 0.9330, *p* = 0.4513; W = 0.8895, *p* = 0.0742; W = 0.8219, *p* = 0.0648; W = 0.8614, *p* = 0.1522; W = 0.9458, *p* = 0.0662; W = 0.8081, *p* = 0.0784; W = 0. 9120, *p* = 0.0579). **(E)** Infectivity of the culture supernatants after neutralization with the anti-S antibody, determined by measuring SARS-CoV-2 RNA levels in hepatocyte culture supernatants using RT-qPCR, expressed as copies/mL. As a control, free SARS-CoV-2 (Wuhan strain, MOI 0.01) was preincubated with anti-S or anti-SB antibodies and subsequently used to infect hepatocytes to assess its replication capacity. Ctrl: untreated hepatocytes. Wh: hepatocytes directly infected with SARS-CoV-2. Wh + anti-S: direct infection after preincubation with anti-Spike antibody. Wh + anti-SB: direct infection after preincubation with control antibody. CS-NI: hepatocytes treated with supernatants from non-infected adipocytes. CS-Wh: hepatocytes treated with supernatants from infected adipocytes. CS-Wh + anti-S/CS-Wh + anti-SB: CS-Wh preincubated with anti-Spike or control antibody, respectively. Non-infected (NI). Days post-infection (DPI). Days post-stimulation (DPS). Control (Ctrl). Culture supernatants (CS). Non-significant (NS). Scale bar: 25 µm. Data are presented as mean ± SD from four independent biological replicates, each performed in technical triplicate. ****p < 0.0001 compared to Ctrl. ^####^*p* < 0.0001.

To determine whether adipocyte-derived soluble factors or infectious viral particles themselves drive hepatocyte lipid droplet accumulation, we pre-incubated culture supernatants from SARS-CoV-2–infected adipocytes with either an anti–spike glycoprotein antibody (anti-S) or a non-specific control, anti-snakebite (anti-SB) for one hour (**Figure 3A**). Neutralization with anti-S restored hepatocyte mean fluorescence intensity and size to basal levels, indicating that the effect dependent on spike-mediated viral entry and/or replication vi In contrast, anti-SB had no impact (**Figures 3B–D**). Consistent with these findings, pre-treatment of the infected adipocyte supernatants with anti-S, but not anti-SB, also blocked SARS-CoV-2 replication in hepatocytes (**Figure 3E**) and similarly prevented direct infection at an MOI of 0.01. Together, these data indicate that SARS-CoV-2 progeny released from adipocytes is associated with lipid droplet accumulation in hepatocytes; however, we cannot exclude the possibility that residual infectious SARS-CoV-2 present in the secretome contributed directly to hepatocyte infection.

### Replication-competent SARS-CoV-2 in adipocyte supernatants drives hepatic lipid accumulation

**Figure 4:**
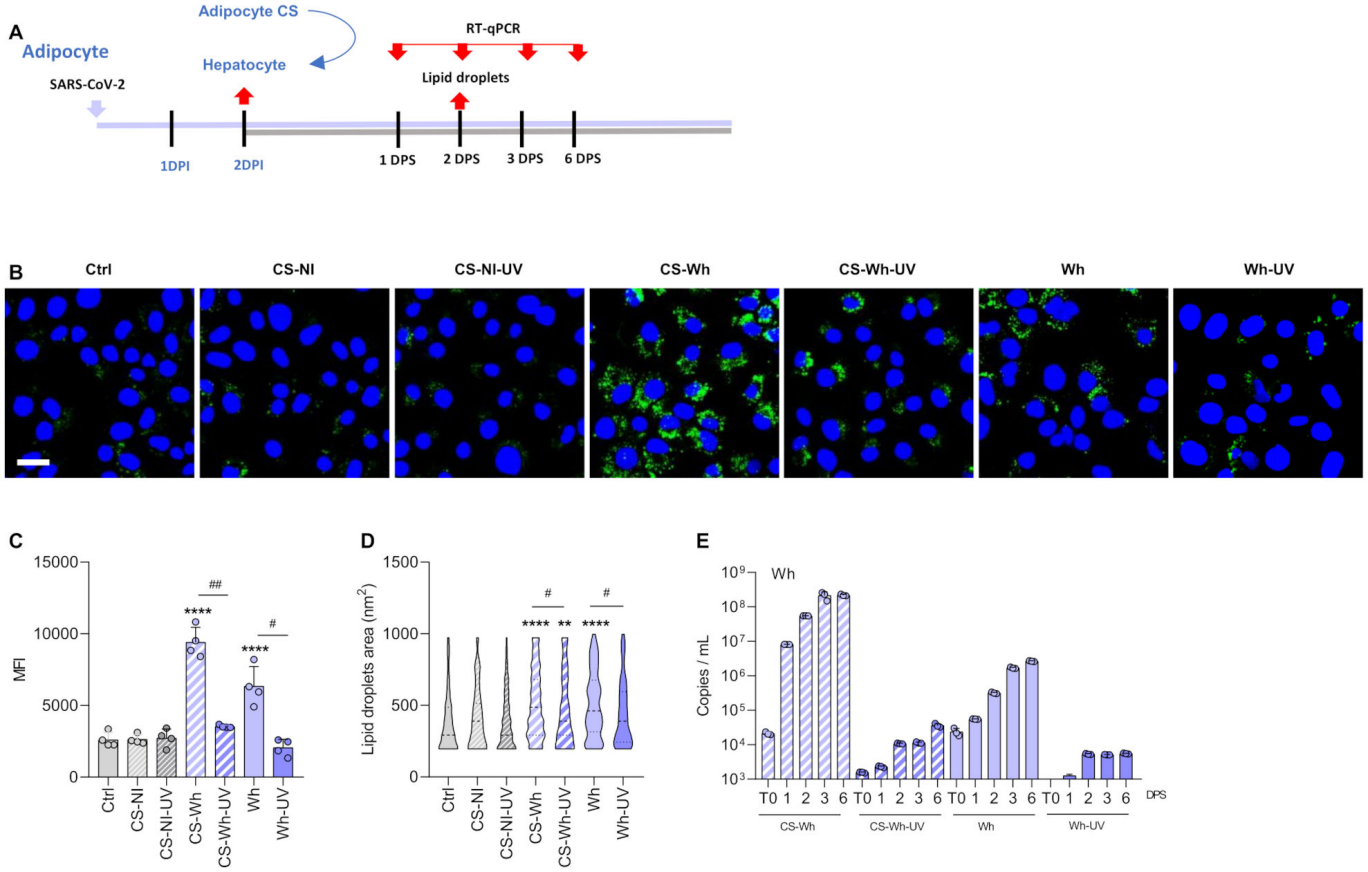
UV-C inactivation abolishes the ability of supernatants from SARS-CoV-2–infected adipocytes to induce lipid droplet accumulation in hepatocytes. **(A)** Schematic representation of the experimental timeline. Adipocytes were infected, and supernatants were collected at 2 DPI. The culture supernatants were then UV-C inactivated and used to stimulate hepatocytes. Lipid droplets were assessed 2 DPS, and viral replication was measured at 1, 2, 3, and 6 DPS **(B)** Representative images of lipid droplets in hepatocytes stimulated with UV-C–inactivated culture supernatants from SARS-CoV-2 (Wuhan strain)–infected adipocytes. Lipid droplets were stained with Bodipy 493/503. **(C, D)** Quantification of lipid droplets shown in panel B: **(C)** mean fluorescence intensity (W = 0.8119, *p* = 0.1252; W = 0.8119, *p* = 0.1253; W = 0.9720, *p* = 0.8541; W = 0.9398, *p* = 0.6532; W = 0.7816, *p* = 0.0731; W = 0.9529, *p* = 0.7346; W = 0.9198, *p* = 0.5360) and **(D)** lipid droplet area (W = 0.8594, *p* = 0.0671; W = 0.8627, *p* = 0,1567; W = 0.8493, *p* = 0,0718; W = 0.9176, *p* = 0.0846; W = 0.8705, *p* = 0.0692; W = 0.9397, *p* = 0.0862; W = 0.8933, *p* = 0.1451). **(E)** Infectivity of the UV-C–inactivated culture supernatants was assessed by measuring SARS-CoV-2 RNA levels in hepatocyte culture supernatants using RT-qPCR and expressed as copies/mL. Non-infected (NI). As a control, free SARS-CoV-2 (Wuhan strain, MOI 0.01) was inactivated with UV-C and subsequently used to infect hepatocytes to assess its replication capacity. Ctrl: untreated hepatocytes. Wh: hepatocytes directly infected with SARS-CoV-2. CS-NI: hepatocytes treated with culture supernatants from non-infected adipocytes. CS-Wh: hepatocytes treated with culture supernatants from SARS-CoV-2-infected adipocytes. UV: CS-Wh samples after UV-C inactivation prior to treatment of hepatocytes. Days post-infection (DPI). Days post-stimulation (DPS). Control (Ctrl). Culture supernatants (CS). Non-significant (NS). Scale bar: 25 µm. Data are presented as mean ± SD from four independent biological replicates for panels **(C, D)**, and three independent biological replicates for panel **(E)**, each performed in technical triplicate. **p < 0.001, ****p < 0.0001 compared to Ctrl. ^#^ *p* < 0.01, ^##^ *p* < 0.001.

Extending our neutralization studies, we next asked whether viral infectivity itself is necessary for hepatocyte lipid remodeling. To test this, we inactivated culture supernatants from SARS-CoV-2–infected adipocytes with UV-C light before exposing hepatocytes to the media (**Figure 4A**). Unlike untreated supernatants, UV-C–treated media failed to elevate lipid droplet mean fluorescence intensity or size above levels seen with uninfected controls. Similarly, UV-C–inactivated SARS-CoV-2 did not induce droplet accumulation, whereas active virus robustly enhanced lipid droplet formation (**Figures 4B–D**). Confirming complete inactivation, neither free virus nor adipocyte-derived virus in UV-C–treated samples could replicate in hepatocytes (**Figure 4E**). These results demonstrate that productive SARS-CoV-2 infection, not merely viral components or mediators synthesized and released by adipocytes, is required to drive lipid droplet accumulation in hepatocytes.

## Discussion

Obesity may contribute to worse COVID-19 outcomes through several mechanisms, including impaired respiratory mechanics (25), a pro-inflammatory and hypercoagulable metabolic state (26–28), compromised immune responses, increased susceptibility to endotoxemia in the context of obesity and aging (14, 29, 30), and the potential for direct infection of adipose tissue by SARS-CoV-2 (31, 32).

In our model, infected adipocytes acquired a proinflammatory phenotype, as reflected by increased IL-6 secretion and an elevated leptin/adiponectin mRNA ratio, together with lipid remodeling. Importantly, our data indicate that the effect on hepatocytes depends on infectious virus present in the adipocyte supernatant, rather than on soluble adipocyte mediators alone. Therefore, these findings support a local adipose–hepatocyte interaction, but do not establish adipose tissue as a dominant systemic reservoir driving whole-body COVID-19 severity.

Here, using adipocytes derived from MSCs, we found ACE2 expression on the adipocyte surface and showed that active SARS-CoV-2 replication increases lipid droplet size and induces a proinflammatory profile characterized by elevated IL-6 secretion and an increased leptin-to-adiponectin ratio. Although secreted leptin and adiponectin protein levels were not measured, the observed triglyceride accumulation and increased glycerol release indicate adipocyte hypertrophy and altered lipolytic activity, supporting a dysfunctional adipocyte phenotype consistent with changes in leptin/adiponectin mRNA expression. These results are consistent with previous findings using adipocytes derived from the human preadipocyte (SGBS) cell line (14). In line with these findings, transcriptomic studies of SARS-CoV-2-exposed human adipose tissue have reported enrichment of pathways associated with IL-6 signaling (7).

Importantly, the increase in lipid droplet size observed in hepatocytes was associated with infectious SARS-CoV-2 present in the adipocyte culture supernatants; however, we cannot exclude the possibility that residual infectious virus in the secretome contributed directly to hepatocyte infection. Accordingly, neutralization of viral entry into hepatocytes with an anti-S antibody abolished this effect.

Consistently, UV-inactivated supernatants failed to induce lipid droplet accumulation, indicating that viral infectivity is required for these metabolic alterations. Therefore, in this experimental system, we cannot attribute hepatocyte lipid accumulation to adipocyte-derived soluble mediators independently of infectious virus present in the secretome. Accordingly, publicly available single-cell RNA-seq data from liver samples of COVID-19 patients (33) revealed that hepatocytes exhibit transcriptional signatures associated with lipid metabolism (Peroxisome Proliferator-Activated Receptor Alpha (PPARA), Fatty Acid Binding Protein 1 (FABP1), and Microsomal Triglyceride Transfer Protein (MTTP) and lipid droplet biogenesis (Perilipin (PLIN)2 and PLIN5), supporting a model in which the virus infection directly reprograms host cellular metabolism to facilitate replication. Hepatocyte transcriptional signatures associated with lipid metabolism and lipid droplet biogenesis, consistent with the possibility that SARS-CoV-2 infection alters hepatocyte metabolic pathways during infection. However, the intracellular mechanisms responsible for lipid accumulation were not directly addressed in the present study.

In conclusion, SARS-CoV-2–infected adipocytes exhibited a proinflammatory and lipid-altering phenotype, while culture supernatants from infected adipocytes promoted hepatocyte lipid accumulation in a manner dependent on viral infectivity. These findings identify a proof-of-principle adipose–hepatocyte crosstalk mechanism *in vitro*. They should not be interpreted as evidence that adipose tissue is a primary respiratory reservoir or that the adipocyte-liver axis alone explains COVID-19 severity in patients. Rather, they suggest a possible mechanism by which pre-existing metabolic dysfunction could be worsened during infection. Also our results suggest that adipose-liver interactions may shape the outcome of viral coinfections, particularly in patients with pre-existing metabolic or liver conditions.

This study has some limitations. Because Huh7.5 is a hepatoma cell line harboring a defective RIG-I pathway, it is highly permissive to viral infection but does not fully reflect innate antiviral signaling in primary hepatocytes; therefore, this model should be interpreted as a proof-of-principle system and validated in additional hepatocyte models.

A further limitation is that only the Wuhan SARS-CoV-2 strain was evaluated. This restricts direct extrapolation of our findings to currently circulating variants, as Omicron and later lineages have been reported to display altered entry characteristics, including reduced dependence on TMPRSS2 and greater reliance on endosomal/cathepsin-mediated entry in some cellular models, together with changes in ACE2 binding affinity. Accordingly, our results should be interpreted as strain-specific and future studies should assess whether the observed adipocyte and hepatocyte effects are conserved across emerging variants. Finally, the intracellular mechanisms responsible for lipid accumulation in infected hepatocytes were not directly investigated. Further studies are needed to determine whether this phenotype is driven by altered lipid uptake, synthesis, oxidation, or export.

In addition, this study was performed entirely *in vitro* and does not allow us to determine whether SARS-CoV-2 induces hepatic steatosis *de novo* in patients or instead exacerbates pre-existing MAFLD. We also did not assess immune-cell infiltration, systemic inflammatory mediators, or clinical correlations in COVID-19 cohorts; therefore, the immunological and clinical relevance of these findings should be interpreted with caution.

## Data Availability

The raw data supporting the conclusions of this article will be made available by the authors, without undue reservation.
